# Supplementary material for: Optical mapping of biological water in single live cells by stimulated Raman excited fluorescence microscopy
Source: Nat Commun. 2019 Oct 18;10:4764. doi: 10.1038/s41467-019-12708-2 (PMC6802100; doi:10.1038/s41467-019-12708-2)
Supplement: Supplementary file 1 — Supplementary Information [file 41467_2019_12708_MOESM1_ESM.pdf]

Supplementary Information

for

Optical mapping of biological water in single live cells by stimulated Raman excited fluorescence microscopy

Shi et al.

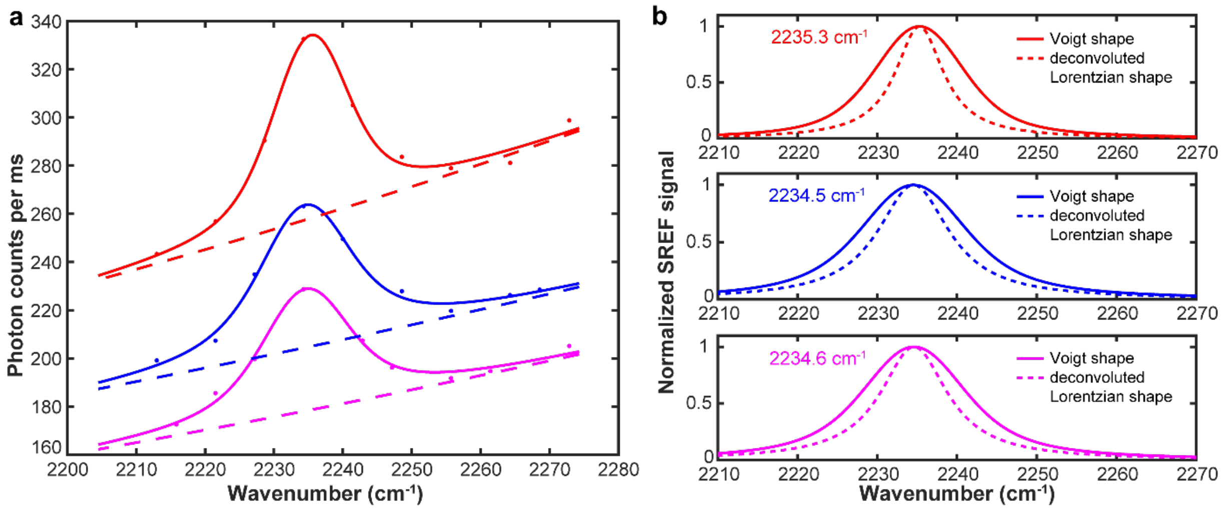

Supplementary Fig. 1 SREF spectra fitting and deconvolution. (a) Model fitting of raw SREF spectra (solid lines with round markers) and corresponding exponential background (dash lines) of 5 μM Rh800 in DMSO. Red, blue and magenta curves represent three replicates. Each data point is the mean of a line scan of 100 pixels. (b) Normalized SREF peaks (after background deduction) as Voigt shape (solid lines) and Lorentzian shape (dash lines) after laser broadening deconvolution. All spectra in Fig. 2 are the deconvoluted Lorentzian profiles using averaged results of three replicates.

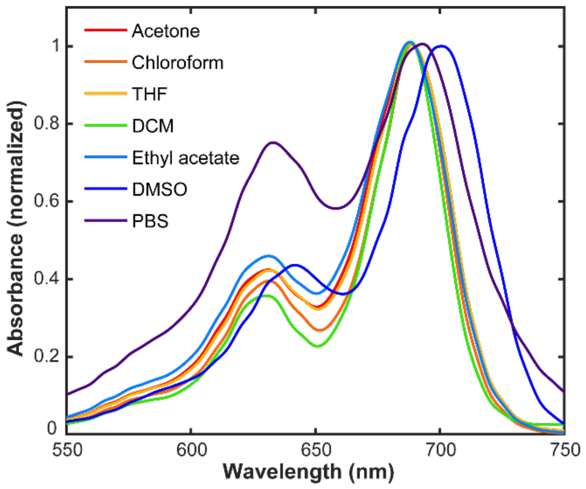

Supplementary Fig. 2 Absorption spectra of Rh800 in different solvents. Acetone (red), chloroform (orange), tetrahydrofuran (gold), dichloromethane (green), ethyl acetate (baby blue), DMSO (blue), water (purple).

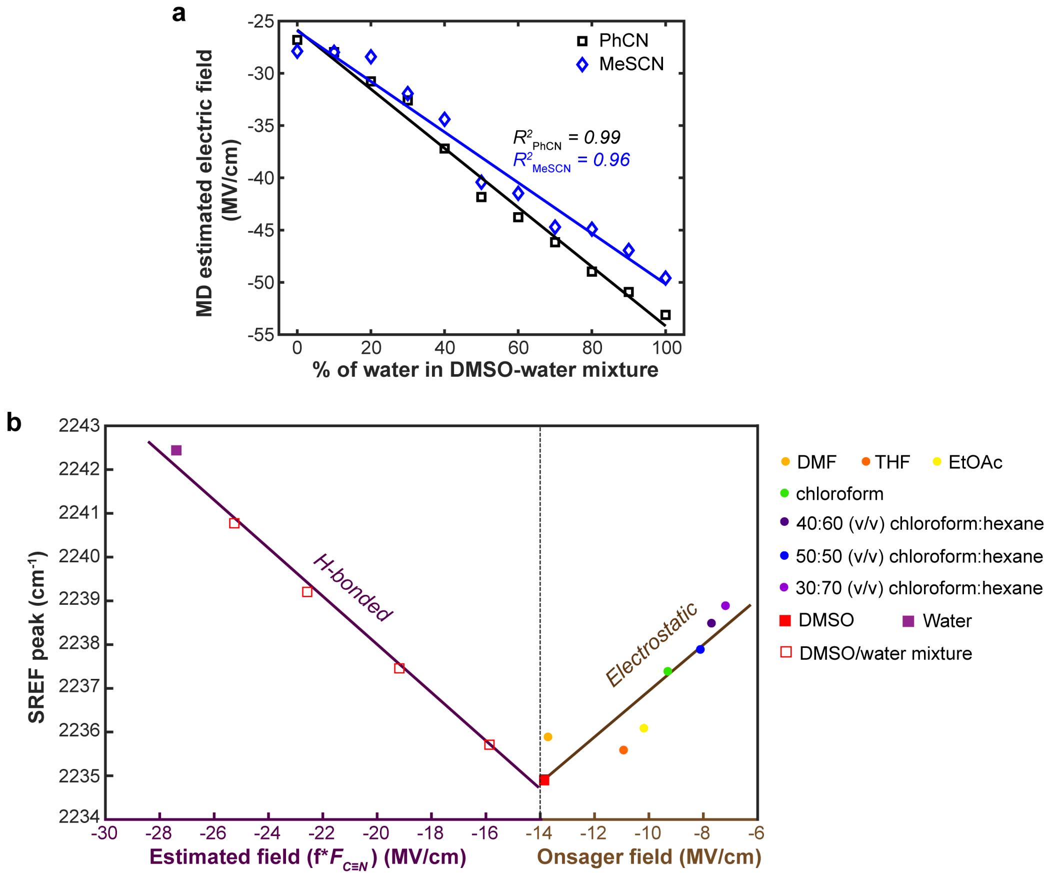

Supplementary Fig. 3 Opposite frequency-shift trends for H-bonding and electrostatic effects. (a) Linear correlation for electric field and water percentage (v/v) in the DMSO-water mixture for nitriles of PhCN and MeSCN. MD estimated electric field data for PhCN and MeSCN were adopted from literature[1]. (b) V-shaped SREF peak frequency and field correlation. The estimated electric field ($f * F_{\mathrm{C\equiv N}}$, f is a correction factor) for Rh800 in DMSO/water mixture was scaled from black curve (PhCN) in (a). Fit line for H-bonded condition is $\bar{\nu}_{\mathrm{C\equiv N}} = -0.55 \cdot f * F_{\mathrm{C\equiv N}} + 2227$, ($R^2 = 0.99$). Fit line for Non-H-bonded condition is $\bar{\nu}_{\mathrm{C\equiv N}} = 0.53 \cdot F_{\mathrm{C\equiv N,Onsager}} + 2242.2$, ($R^2 = 0.84$).

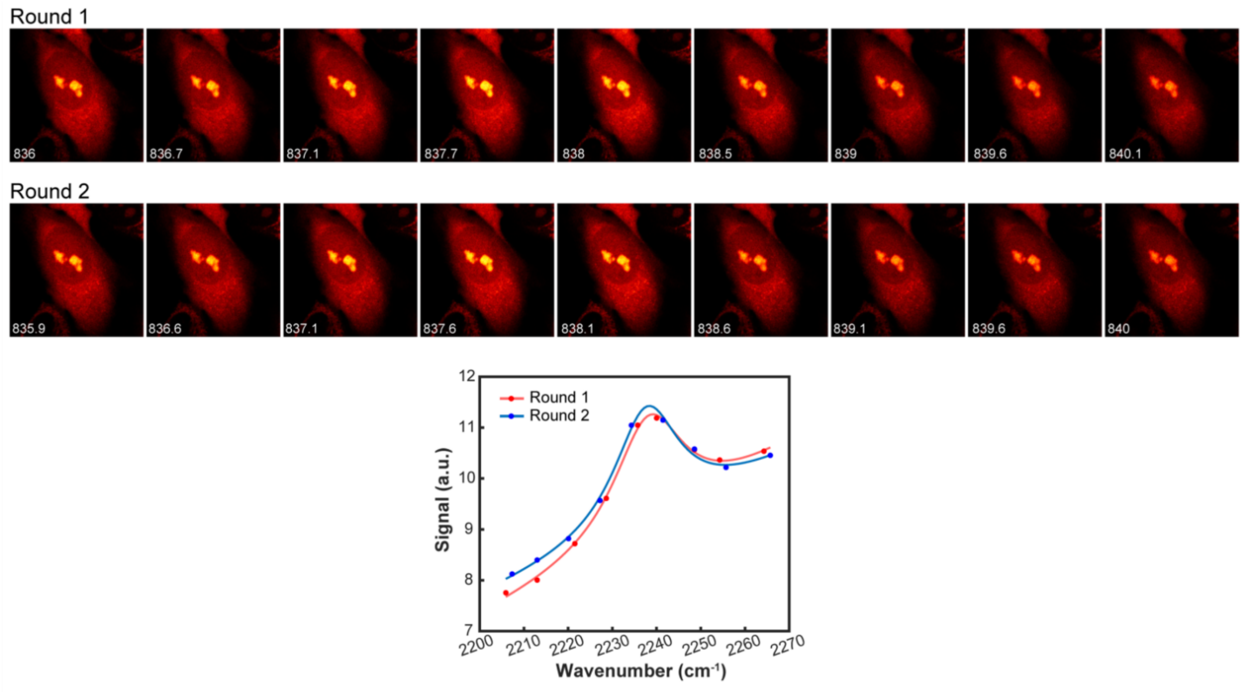

Supplementary Fig. 4 Photobleaching characterization during SREF spectral sweeping. Two rounds of 9-point SREF spectral mapping were acquired with P_{pump}=10 mW and P_{Stokes}=12 mW with 10-µs pixel dwell time. Pump beam wavelength for each image is given at the lower left corner. No obvious photobleaching can be observed based on reproducible spectra and consistent cell patterns of two rounds.

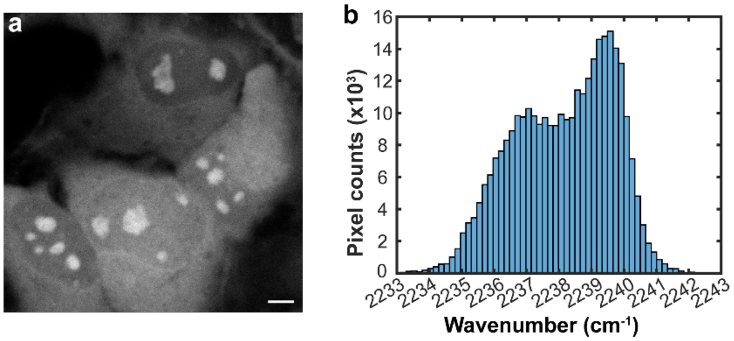

Supplementary Fig. 5 SREF peak frequency histogram of live cells in Fig. 3. (a) A representative image (Pump = 836 nm) of 9 sequential images with Pump wavelength scanning across vibrational resonance. (b) Histogram of nitriles peak for all pixels in (a).

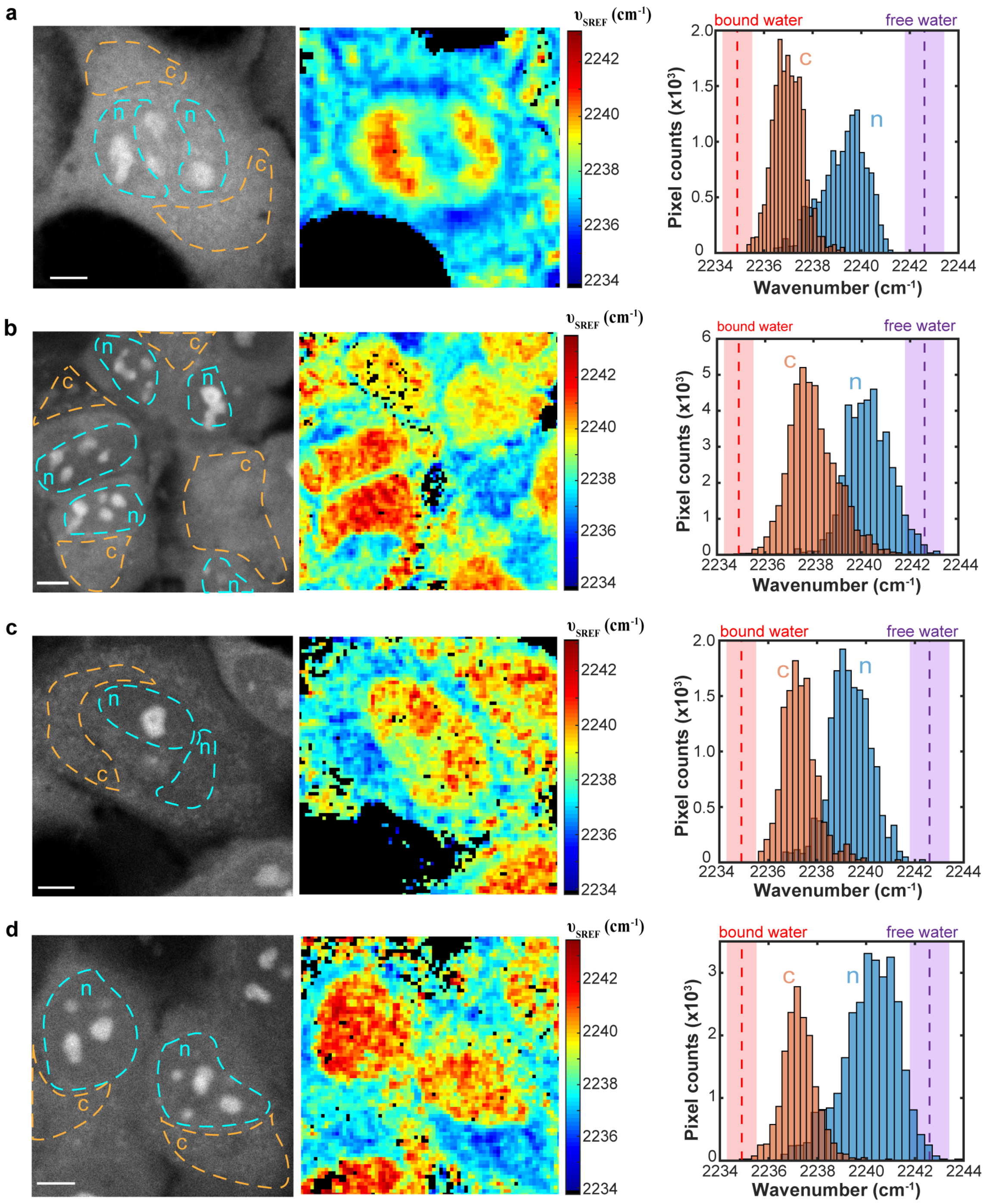

Supplementary Fig. 6 SREF frequency mapping in Rh800 stained live HeLa cells. (left) A representative image (pump=836 nm) of 9 sequential images with pump wavelength scanning across vibrational resonance. (middle) SREF peak frequency mapping images. (right) Nitrile peak frequency histogram of two regions (n for nucleus and c for cytoplasm, marked in left column with dash line) of live HeLa cells. Red and purple dashed lines with shadows show statistics of minimum and maximum SREF peak frequencies (mean±s.d.) from 21 cells, representing $\bar{\nu}_{C\equiv N}$ in bound-water and free-water states, respectively. (a-d) Four more sets of SREF peak mapping show

similar results as Fig. 3a-c indicating consistent observation of higher SREF peak frequency (blue-shifted) in nucleus compared to cytoplasm. Scale bars, 5 μm. Pixel dwell time, 10 μs.

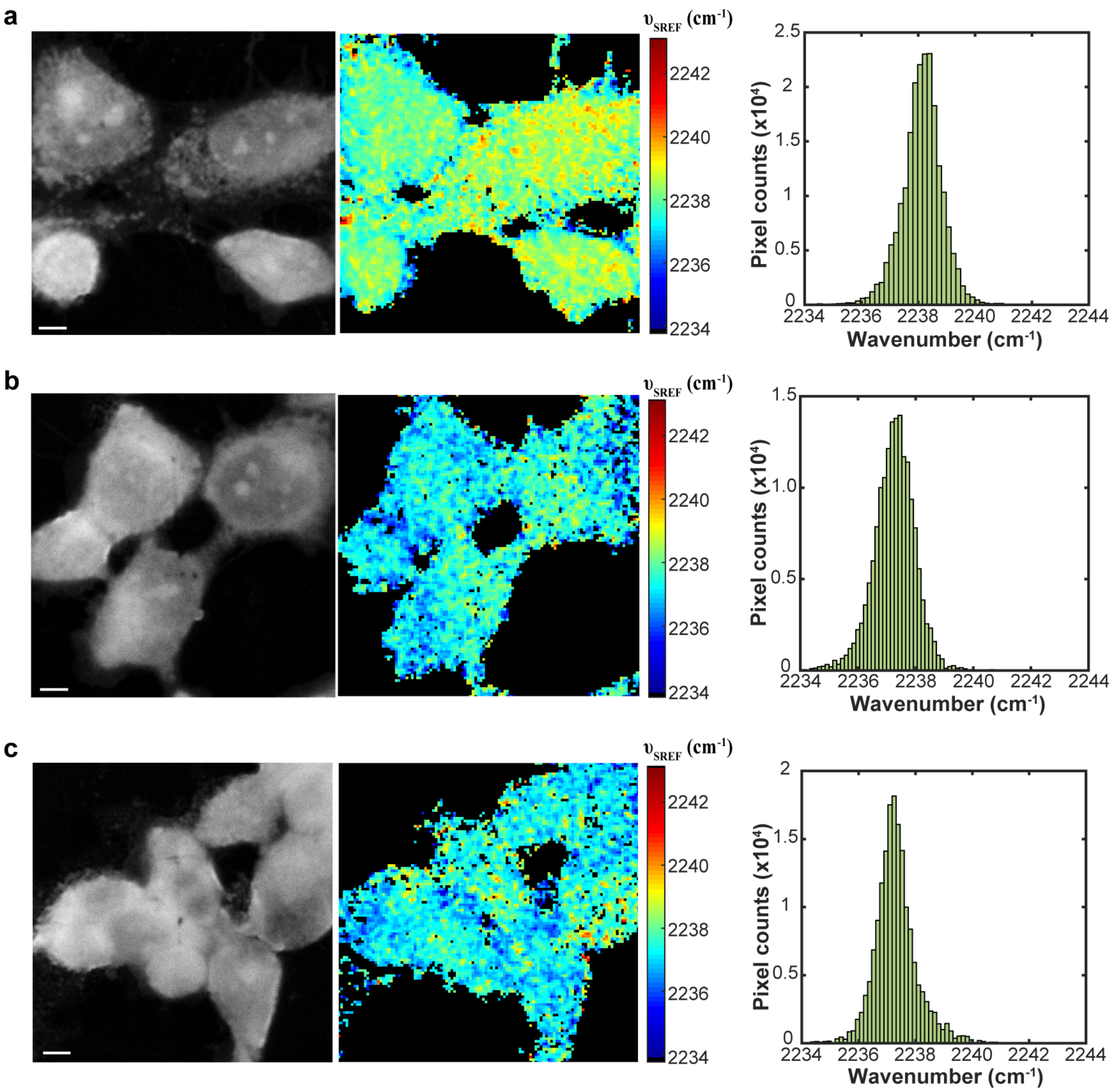

Supplementary Fig. 7 SREF frequency mapping in Rh800-stained ethanol-dried HeLa cells. (left) A representative image (pump = 836 nm) of 9 sequential images with pump wavelength scanning across vibrational resonance. (middle) SREF peak frequency mapping images. (right) peak-frequency histogram of all pixels in the middle column. (a-c) Three more sets of SREF peak mapping show similar results as Fig.3e-g with homogeneous peak distribution within cells. Scale bars, 5 µm. Pixel dwell time, 10 µs.

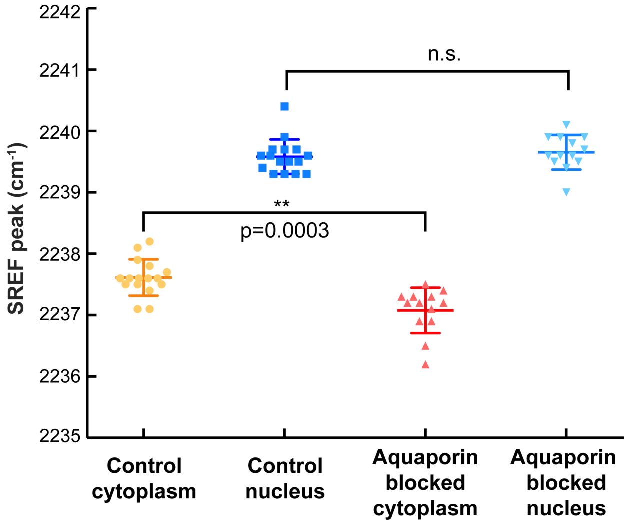

Supplementary Fig. 8 Blocking water exchange in live HeLa cells causes $\bar{\upsilon}_{C\equiv N}$ shift in cytoplasm. Nitriles peak frequency (mean ± s.d., n=16) for the control cells is 2237.7 ± 0.3 cm^{-1} and 2239.9±0.3 cm^{-1} inside cytoplasm and nucleus, respectively. Nitriles peak frequency (mean±s.d., n=13) for the aquaporin-blocked cells is 2237.1 ±0.3 cm^{-1} and 2239.7±0.3 cm^{-1} inside cytoplasm and nucleus, respectively. For aquaporin blocked sets, HeLa cells were incubated with 200-µM $HgCl_2$ for 30 min. Cell were all stained with 2-µM Rh800 for 30 min. Shown as, two-sided unpaired t-test for cytoplasm gives p=0.0003 and is not significant for nucleus. Double asterisks indicate p<0.001. Error bars represent standard deviations.

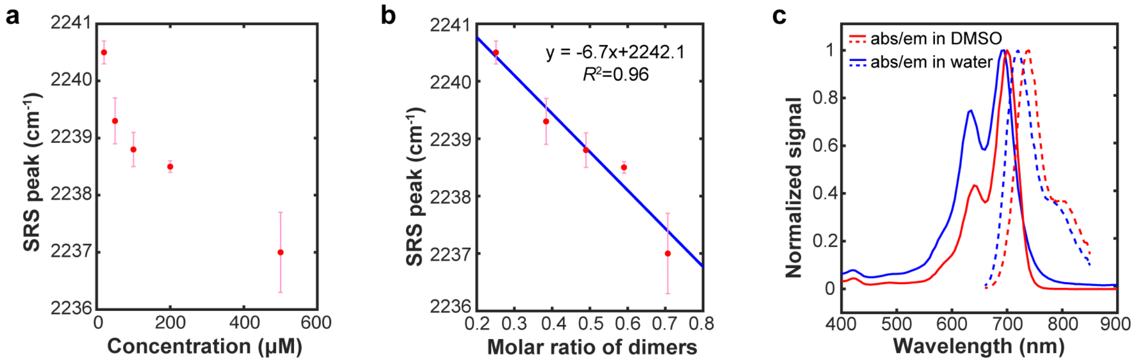

Supplementary Fig. 9 Aggregation effect for Rh800 perturbs $\bar{\nu}_{C\equiv N}$ measured by SRS. (a) SRS peak of Rh800 nitrile mode red-shift with increasing concentration in water. Small amount (<1% v/v) of DMSO were also added to improve dye solubility. (b) Linear correlation of SRS peak with the dimer molar ratio calculated by the equilibrium constant. The intercept which is 2242.1 cm^{-1} represents the fitted SRS peak for nitrile mode of monomer Rh800 in water. (c) Absorption and fluorescence emission spectra of Rh800 in DMSO and water. Dimer formation in water influences the shape of absorption spectra but not of emission spectra. More details and the calculation process can be found in Supplementary Table 3 and Supplementary Note 1. Error bars represent standard errors of regression under 90% confidence level.

Solvent	n_d	Dielectric constant	Dipole moment (Debye)	Cavity radius a^3 (Å^3)	Electrical field (MV/cm)	SREF peak (cm^{-1})	SREF FWHM (cm^{-1})
dimethyl sulfoxide	1.50	47.00	5.5978	164	-13.86	2234.9	8.8
tetrahydrofuran	1.50	7.52	5.5978	164	-10.93	2235.6	7.8
DMF	1.50	38.25	5.5978	164	-13.71	2235.9	11.4
ethyl acetate	1.50	6.02	5.5978	164	-10.18	2236.1	5.2
acetone	1.50	21.01	5.5978	164	-13.10	2236.8	5.1
chloroform	1.50	4.81	5.5978	164	-9.31	2237.4	6.5
DCM	1.50	10.36	5.5978	164	-11.81	2237.5	5.1
50% (v/v) chloroform + 50% (v/v) hexane	1.50	3.70	5.5978	164	-8.11	2237.9	6.6
40% (v/v) chloroform + 60% (v/v) hexane	1.50	3.41	5.5978	164	-7.70	2238.5	7.6
30% (v/v) chloroform + 70% (v/v) hexane	1.50	3.09	5.5978	164	-7.18	2238.9	9.7

Supplementary Table 1. SREF spectral data for C≡N band of Rh800 in aprotic solvents and mixtures. Samples in DMSO, DMF, THF, ethyl acetate were at 10 µM concentration and samples in acetone, chloroform, DCM and chloroform/hexane were at 20 µM concentration. SREF peak position and FWHM are obtained with curve fitting as described in Methods.

Volume percentage of water in DMSO-water mixture (%)	SREF peak (cm^{-1})	FWHM (cm^{-1})
0	2234.9	9.1
20	2235.7	11.5
40	2237.5	10.8
60	2239.2	10.5
80	2240.8	11.0
100	2242.4	8.8

Supplementary Table 2. SREF spectral data for C≡N band of Rh800 in DMSO/water binary mixture. Samples in DMSO, 20% water, 40% water, 60% water were at 10 µM concentration and samples in 80% water and 100% water were at 20 µM concentration. SREF peak position and FWHM are obtained with curve fitting as described in Methods.

Total concentration (μM)	[Dimer] (μM)	[Monomer] (μM)	Dimer molar ratio [D]/([D] + [M])	SRS peak (cm^{-1})	peak error (cm^{-1})
500	207	86	0.71	2237.2	0.6
200	74	51	0.59	2238.5	0.1
100	33	34	0.49	2238.8	0.3
50	14	22	0.38	2239.3	0.4
20	4	12	0.25	2240.5	0.2
10	1.43	7.14	0.17	/	/
2	0.092	1.82	0.048	/	/
0.5	0.007	0.487	0.013	/	/

Supplementary Table 3. Rh800 dimer molar ratios and SRS peak frequencies under different concentrations. Monomer and dimer concentrations are calculated as $[\mathrm{Monomer}]_i = \frac{1}{4K_{\mathrm{eq}}}\left(\sqrt{8C_iK_{\mathrm{eq}}+1}-1\right), [\mathrm{Dimer}]_i = \frac{C_i}{2} - \frac{1}{8K_{\mathrm{eq}}}\left(\sqrt{8C_iK_{\mathrm{eq}}+1}-1\right)$, where C_i is the total Rh800 concentration and K_{eq} is the dimerization equilibrium constant which is 28000. SRS peak position is fitted by Lorentzian function. Peak error is the standard error of regression under 90% confidence level.

Supplementary Note 1. Aggregation effect

Aggregation is well known to take places for cationic dyes in water as a concentration-induced effect. For example, a so-called H-dimer (hypsochromically-shifted absorption for dimer) is typically formed in aqueous solution for many oxazine dyes.[2] Such aggregation effect is also observed for Rh800 and has been characterized by steady-state and ultrafast spectroscopy in literature.[3]

Briefly, a concentration-dependent monomer (M) and dimer (D) equilibrium will form in water for Rh800. As a result, the SRS peak frequency is found to shift with concentration (Supplementary Fig. 9a, Supplementary Table 3). With increased concentration, the SRS nitrile peak of Rh800 will red-shift to lower frequency. This trend may indicate the dimer formation will perturb the hydrogen bonding of the nitrile with water. Using the equilibrium constant of dimerization (M+M→D, K_{eq}=28000) fitted by absorption spectra on different concentrations[3], we can calculate the dimer molar ratios under different concentrations as listed in Supplementary Table 3. By fitting the SRS peak frequency with the dimer molar ratio, a linear correlation is found as shown in Supplementary Fig. 9b. Hence the intercept at 2242.1 cm^{-1} represents the fitted SRS peak for pure monomer Rh800 in water. As an internal control, this SRS peak frequency is in accordance with the SREF peak measured in water as 2242.4 cm^{-1}.

Different from the undesirable concentration-dependent SRS frequency shift of Rh800, we did not observe much aggregation effect on SREF frequency. A saturated Rh800 aqueous solution (~80 $\boldsymbol{\mu}M$) showed a similar peak position at 2242.2 cm^{-1} compared to $\bar{\nu}_{\mathrm{C\equiv N},10\ \boldsymbol{\mu}M\ \text{in water}} = 2242.4$ cm^{-1}. Such profound difference between SRS and SREF here is because the dimer does not fluoresce owing to its extremely short excited state lifetime as $\tau_D = 3$ ps[3]. In analogy, the dimer formation in water will only perturb the shape of absorption but not of fluorescence emission (Supplementary Fig. 9c). Overall, the absence of dimer fluorescence helps SREF specifically monitor Rh800 monomers while SRS measures the overall response of dimers and monomers.

Supplementary References

1. Deb, P. *et al.* Correlating nitrile IR frequencies to local electrostatics quantifies noncovalent interactions of peptides and proteins. *J. Phys. Chem. B* **120**, 4034-4046, (2016).
2. Steinhurst, D. A. & Owrutsky, J. C. Second harmonic generation from oxazine dyes at the air/water interface. *J. Phys. Chem. B* **105**, 3062-3072, (2001).
3. Sekiguchi, K., Yamaguchi, S. & Tahara, T. Formation and dissociation of Rhodamine 800 dimers in water: steady-state and ultrafast spectroscopic study. *J. Phys. Chem. A* **110**, 2601-2606, (2006).
